# Interferon-Lambda: A Potent Regulator of Intestinal Viral Infections

**DOI:** 10.3389/fimmu.2017.00749

**Published:** 2017-06-30

**Authors:** Sanghyun Lee, Megan T. Baldridge

**Affiliations:** ^1^Department of Pathology and Immunology, Washington University School of Medicine, St. Louis, MO, United States; ^2^Department of Medicine, Division of Infectious Diseases, Washington University School of Medicine, St. Louis, MO, United States

**Keywords:** interferon-lambda, enteric virus, innate immunity, transkingdom interactions, norovirus, rotavirus, commensal bacteria

## Abstract

Interferon-lambda (IFN-λ) is a recently described cytokine found to be of critical importance in innate immune regulation of intestinal viruses. Endogenous IFN-λ has potent antiviral effects and has been shown to control multiple intestinal viruses and may represent a factor that contributes to human variability in response to infection. Importantly, recombinant IFN-λ has therapeutic potential against enteric viral infections, many of which lack other effective treatments. In this mini-review, we describe recent advances regarding IFN-λ-mediated regulation of enteric viruses with important clinical relevance including rotavirus, reovirus, and norovirus. We also briefly discuss IFN-λ interactions with other cytokines important in the intestine, and how IFN-λ may play a role in regulation of intestinal viruses by the commensal microbiome. Finally, we indicate currently outstanding questions regarding IFN-λ control of enteric infections that remain to be explored to enhance our understanding of this important immune molecule.

## An Introduction to Interferon-Lambda (IFN-λ) in the Intestine

Animals can mount potent and rapid innate immune responses to invading viruses. The classic signaling pathway by which this response occurs is *via* type I interferons (IFNs), including IFN-beta (IFN-β) and multiple IFN-alphas (IFN-α) (1). When cells sense viral products, type I IFNs are produced, which stimulate transcription of antiviral molecules that act in autocrine and paracrine fashion. However, in the past decade, an important paradigm shift has occurred in how we consider the compartmentalization of viral responses into systemic versus mucosal responders, driven in large part by the discovery of type III IFNs, or IFN-λ.

**Figure 1:**
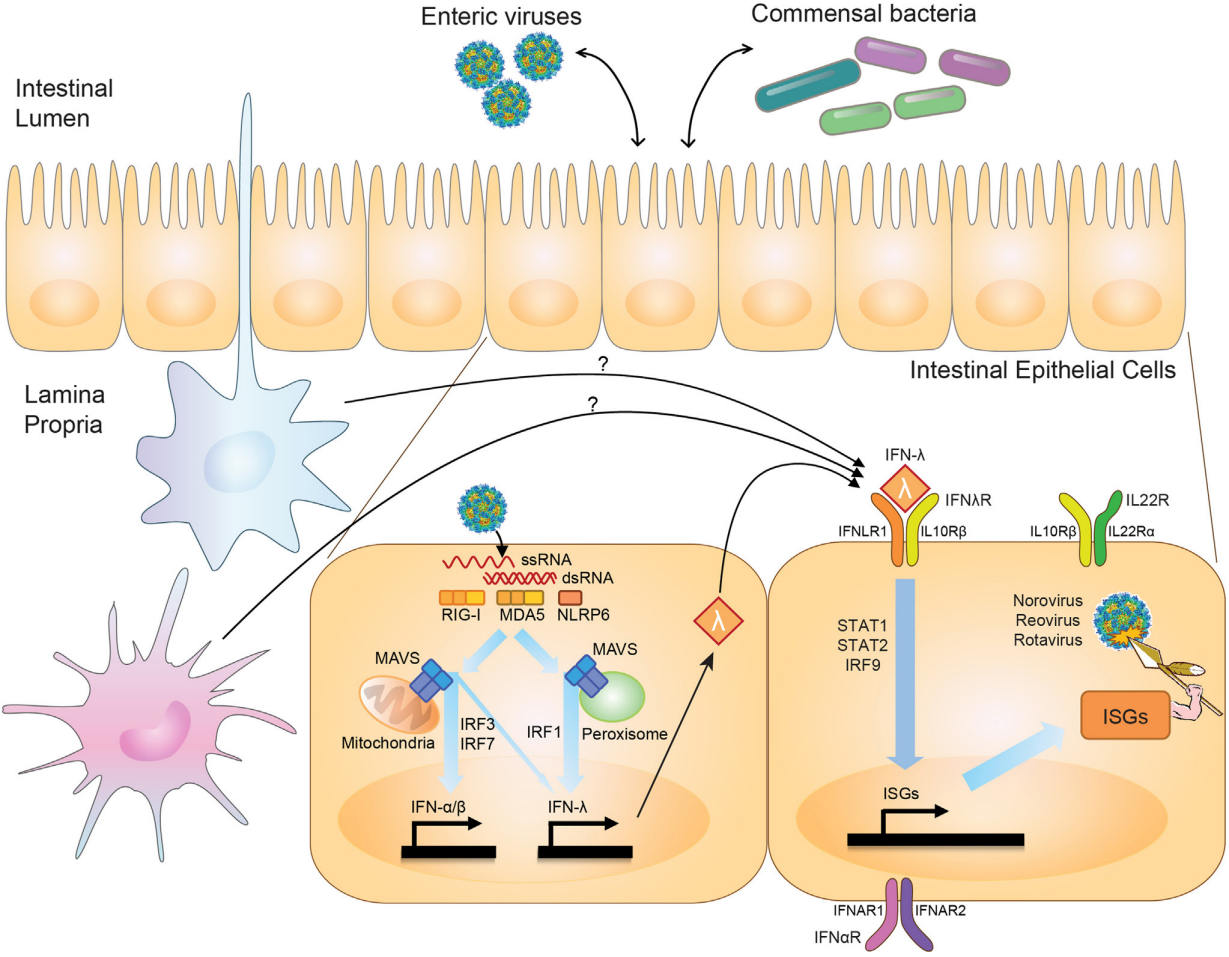
Effects of interferon-lambda (IFN-λ) on viruses in the intestine. Upon intestinal viral infection, viral RNA is sensed by pattern-recognition receptors, RIG-I, MDA5, and NLRP6, which signal through mitochondria- or peroxisome-associated MAVS to stimulate transcription of type I and III IFN by IRF3/IRF7 and IRF1. IFN-λ is produced by intestinal epithelial cells (IECs) and possibly immune cells in the intestine. IFN-λ signaling through the IFN-λ-receptor (IFNλR) on IECs stimulates production of antiviral effectors, or interferon-stimulated genes (ISGs), *via* STAT1/STAT2/IRF9-mediated transcription. IFN-λ thus serves to regulate viral levels in the intestine. IFN-λ can interact with IL-22, whose receptor is expressed on IECs (IL22R), to coordinately regulate viral infection, and in some settings may also interact with type I IFNs, which signal through IFNαR. IFN-λ has also been shown to play a role in influencing interactions between commensal bacteria and enteric viral pathogens.

First described in 2003 (2, 3), the IFN-λ family of cytokines includes up to four members in humans, dependent on genetic polymorphisms (4, 5), and two functional orthologs in mice (6, 7). The family, likely arising from a common ancestral fish IFN gene that gave rise to both type I and III IFN families, is conserved to chickens (8, 9). The type III IFNs are under positive selection, with long-term persistence of duplicate copies suggesting a critical biological role for type III IFNs independent from type I IFNs (9). Pattern-recognition receptors, including RIG-I and MDA5, detect viruses and induce type I and III IFNs *via* MAVS and IRF3/IRF7 signaling (10–12) (Figure 1). IRF1 plays a unique role in type III IFN induction, however, being specifically stimulated by peroxisome-associated MAVS in contrast to mitochondrial-associated MAVS, which better induces type I IFNs (13). Intestinal epithelial cells (IECs) produce type III IFNs with *in vivo* viral infection (14–16). However, leukocytes generate IFN-λ *in vitro* (10, 17), and intestinal eosinophils (18) and plasmacytoid dendritic cells (pDCs) (19) can produce IFN-λ *in vivo*, suggesting the possibility of additional cellular IFN-λ contributors.

While the antiviral programs induced by type I and type III IFNs exhibit substantial overlap (20–22) (Figure 1), a critical difference between the two is the cell types they affect secondary to receptor expression. The IFN-λ receptor consists of IFNLR1 and IL10Rβ. While the receptor for type I IFNs, IFNAR1, is expressed broadly on the majority of cell types, IFNLR1 exhibits a much more restricted pattern of expression (23). In the intestine, IFNLR1 is expressed preferentially on IECs, allowing for a compartmentalized response to viruses infecting at this mucosal surface (24, 25). While IFNLR1 expression has also been reported on NK cells, T cells, B cells, and pDCs (26–30), no role has been found for these cells in IFN-λ-mediated antiviral responses. Type I IFNs, on the other hand, are critical for preventing a virus from moving past this initial epithelial barrier into systemic tissues (24, 25, 31). The host may benefit by inducing specific and local barrier defenses at a site commonly exposed to pathogens *via* IFN-λ signaling, and thus avoid potentially detrimental systemic inflammatory responses by type I IFNs. Many autoimmune diseases, as well as the congenital interferonopathies, are secondary to excessive type I IFN activity (32, 33).

There is an ever-expanding set of roles being discovered for IFN-λ signaling, from control of viral infections in liver (34), lung (35), and brain (36), to regulation of non-infectious diseases like inflammatory bowel disease (37) and cancer (38). Many of these intriguing advances are addressed elsewhere in this Frontiers in Immunology topic, “Interferon-λs: New Regulators of Inflammatory Processes.” Here, however, we will focus exclusively on the regulation of enteric viruses by IFN-λ. We review the current literature about IFN-λ-mediated regulation of specific intestinal viruses, discuss interplay of IFN-λ with other cytokines and its regulation of viral–bacterial interactions, and highlight areas ripe for future research enterprises.

## Regulation of Specific Enteric Viruses by IFN-λ

Enteric viruses, including rotavirus (RV), reovirus, norovirus (NoV), and others, generally infect *via* the fecal–oral route, though other transmission routes have been described. As such, the IECs comprising the mucosal barrier of the intestine likely represent the first eukaryotic cells with which an enteric virus interacts. Here, we describe what is known about specific enteric viruses and their relationship with both the intestinal epithelium and IFN-λ (Table 1).

**Table 1:** Interferon-lambda (IFN-λ) interactions with intestinal viruses *in vivo* and *in vitro*.

Virus	Strain	*In vivo* phenotypes	*In vitro* phenotypes	Reference
Rotavirus (RV)	EDIM	* Mice lacking *Ifnlr1* in all cells exhibit increased viral shedding, intestinal titers, and tissue damage * RV infection induces IFN-λ production in intestinal epithelial cells (IECs) * Treatment with exogenous IFN-λ prevents RV replication in the intestine		(16, 41)
	EW	* Mice lacking *Ifnlr1, Ifnar1*, or *Stat1* in all cells exhibit similar level of viral shedding, intestinal titers		(24)
	Rhesus strain of rotavirus	* Mice lacking *Ifnlr1, Ifnar1*, or *Stat1* in all cells exhibit increased level of viral shedding, intestinal titers		(24)
	Ito, Wa		* Human RV infection induces IFN-λ expression in human intestinal enteroids * Treatment with exogenous IFN-λ inhibits RV replication in enteroids * Blocking endogenous IFN-λ has no effect on viral production	(43)
Reovirus	Type 3 Dearing	* Adult mice lacking *Ifnlr1* in all cells exhibit higher viral shedding of reovirus * Suckling mice lacking *Ifnlr1* in all cells exhibit higher viral shedding and tissue titers of reovirus, increased tissue damage and severe mortality * Mice lacking *Ifnlr1* exhibit higher reovirus infection in IECs, while mice lacking *Ifnar1* exhibit higher infection in lamina propria cells		(14)
	Type 1 Lang	* Mice lacking *Ifnlr1* in all cells or only in IECs exhibit higher viral shedding and intestinal titers of reovirus		(50)
Norovirus	CR6	* Mice lacking *Ifnlr1* in all cells or only in IECs exhibit higher viral shedding and intestinal titers of persistent murine NoV (MNoV) * Treatment with recombinant IFN-λ prevents and cures persistent MNoV infection, dependent on IEC expression of *Ifnlr1* * NoV dependence on the commensal microbiome for infection is absent in mice lacking *Ifnlr1*		(50, 64, 93)
			* Replication of transfected human NoV RNA is sensitive to IFN-λ treatment, but does not induce IFN-λ expression	(59)
Enterovirus	EV71		* Enterovirus 71 induces IFN-λ expression in human IEC line	(72)
Parvovirus			* Canine parvovirus is more sensitive to IFN-λ than type I IFN	(73)
Coronavirus	CV777 LNCT2		* Porcine epidemic diarrhea virus is sensitive to IFN-λ when cultured in a porcine IEC line	(74)

### Rotavirus

Rotaviruses are double-stranded RNA viruses of the *Reoviridae* family and a major cause of severe diarrhea in children worldwide (39). RV infection exhibits a preferential tropism for IECs of the small intestinal villi in humans and mice (40). Several groups have reported antiviral effects of IFN-λ against RV in mouse models (16, 24, 41). Infection by a murine RV, EDIM-RV, induces IFN-λ in the small intestine, and endogenous IFN-λ suppresses intestinal viral replication (16, 41). RIG-I and MDA5 are required for type I IFN production by IECs during RV infection (42); induction pathways for IFN-λ have not been reported. IECs produce the majority of IFN-λ, consistent with the viral IEC tropism (16). Pretreatment with exogenous IFN-λ effectively prevents EDIM-RV replication in the small intestine and colon (41). However, a recent study demonstrated that a homologous murine strain of RV, EW–RV, is largely IFN-λ-insensitive, even though EW-RV is originally derived from EDIM-RV (24). This study also showed that a heterologous rhesus strain of rotavirus (RRV) is, in contrast, highly sensitive to both IFN-α/β and IFN-λ, even though EW-RV and RRV infection both significantly induce IFN-α/β and IFN-λ production during infection (24). The reason for this discrepancy between strains is still unclear, though recently, a human RV study using human intestinal enteroids provided some hints regarding the source of this strain complexity (43). In this study, human RV infection in enteroids indeed induced IFN-λ and interferon-stimulated genes (ISGs). However, blocking IFN-λ signaling did not have any effect on viral growth. Since RV has multiple functional proteins for immune evasion (e.g., NSP1, NSP3, and VP3) (44), the effect of IFN-λ may be limited by these viral genes, and EW–RV may utilize evasion strategies to overcome IFN responses. Thus, interactions between RV and IFN-λ in the intestine are influenced by multiple host and viral factors.

### Reovirus

Although reoviruses are also in the *Reoviridae* family, in contrast to RVs, they are not generally associated with serious human disease. Recently, however, they have been implicated in the pathogenesis of celiac disease, suggesting the possibility of a previously overlooked role as an environmental inflammatory trigger (45). Importantly, reoviruses have been used as a tractable experimental system for studies of viral pathogenesis in newborn mice (46). Reoviruses induce type I and III IFNs in a MAVS-dependent fashion (13, 15, 47), likely *via* RIG-I- and MDA5-mediated sensing (48, 49). Since these viruses exhibit a wide cellular tropism and a low degree of species specificity, reovirus infection of the mouse intestine is sensitive to both IFN-α/β and IFN-λ (14). In adult mice, endogenous IFN-λ inhibits reovirus strain Type 3 Dearing replication in the intestine, and reovirus replicates exclusively in IECs of *Ifnlr1-*deficient mice (14). By contrast, IFN-α/β inhibits reovirus replication in the intestine, but acts specifically on cells in the lamina propria. Another study using reovirus strain Type 1 Lang showed that endogenous IFN-λ inhibits reovirus replication in the mouse small intestine and that IFN-λ-receptor expression in IECs is critical for this antiviral activity (50). Therefore, IFN-λ in the intestine controls reovirus replication in IECs, but IFN-α/β also coordinately controls reovirus infection in non-IEC cell types in the intestine.

### Norovirus

Noroviruses are positive sense non-enveloped RNA viruses in the *Caliciviridae* family (51). In humans, they are the most common cause of epidemic gastroenteritis and are a significant contributor to childhood mortality worldwide (52, 53). In addition to causing acute symptomatic infections characterized by vomiting and diarrhea, they can persist in both immunocompetent (54) and immunocompromised individuals (55), who can potentially seed future epidemics (56). Until quite recently, human NoV has been impractical to culture *in vitro* (57, 58) and lacked a robust small animal model. Secondary to these challenges, the role of IFN-λ in control of human NoV *in vivo* is unknown. *In vitro*, human NoV RNA replication and virus production, after transfection of stool-isolated RNA into mammalian cells, is sensitive to treatment with type I and III IFNs (59). However, in this system, NoV RNA replication does not induce IFNs or respond to neutralization of type I or III IFNs (59). Whether this reflects the *in vivo* effects of NoV infection remains to be seen.

The discovery of murine NoV (MNoV) (60), which is readily culturable (61) and can be studied *in vivo*, facilitated exploration of the interactions between NoV and the host immune system (62). IFNs have long been known to be important in MNoV regulation, as the virus was originally isolated from and causes severe disease and death in *Stat1-*deficient mice (60, 63). Type I and II IFNs both control acute, systemically spreading strains of MNoV [recently reviewed in Ref. (62)]. By contrast, type I and II IFNs are dispensable for intestinal regulation of persistent strains of MNoV (64), which replicate robustly in the colon and are shed at high levels in the stool (65). Instead, for persistent MNoV, IFN-λ plays a critical regulatory role. Endogenous IFN-λ controls intestinal viral replication and shedding, demonstrated by increased shedding in *Ifnlr1-*deficient mice. In addition, exogenous IFN-λ prevents and cures persistent MNoV infection in wild-type and *Rag1-*deficient mice (64). Thus, IFN-λ represents an example of sterilizing innate immunity. Because myeloid and B cells, which support MNoV replication *in vitro* (57, 61), and IECs, the target cells of IFN-λ for MNoV clearance (50), are distinct, it remains to be determined whether *in vivo* IFN-λ stimulates an antiviral program in a cell-intrinsic fashion to clear infected IECs, or instead drives production of secondary factors to target infected myeloid or B cells.

Induction of IFN-λ is also important for control of intestinal MNoV. MDA5 is critical for type I IFN responses to MNoV (66); type III IFN responses may be similarly regulated. Nod-like receptor Nlrp6 is another viral RNA sensor that regulates intestinal MNoV levels and plays a role in induction of type I and III IFNs and ISGs in response to infection (67). Activated intestinal intraepithelial lymphocytes have been shown to rapidly stimulate type I and III IFN receptor-dependent upregulation of ISGs in IECs, which correspondingly limits MNoV infection *in vivo* (68). Persistent strains of MNoV may induce lower levels of type I and III IFNs than acute systemic strains, such that avoidance of IFN upregulation may contribute to persistence of some strains (64).

Identifying viral antagonists of host pathways can highlight critical antiviral host pathways. MNoV antagonizes IFNs *via* a protein expressed from ORF4, VF1, which *in vitro* delays upregulation of innate genes including type I IFNs (69). MNoV has also been shown to diminish the host response to infection *via* its protease NS6, which specifically suppresses host ISG translation (70). However, the interactions of these genes with IFN-λ signaling in the intestine have not yet been explored. A final potential viral player of interest is MNoV NS1/2. A single amino acid difference in this gene confers the ability of the virus to persist in the intestine and stool (65). It is a tempting speculation that intestinal viral persistence requires antagonism of IFN-λ, but further studies are needed to determine whether NoV has evolved to avoid the antiviral effects of this signaling pathway.

### Other Enteric Viruses

A limited number of studies have explored the role of IFN-λ in regulation of other enteric viruses. Infection of human enteroids by echovirus 11, but not coxsackievirus B, was shown to induce expression of antiviral ISGs (71), and enterovirus 71 potently induces type I and III IFNs in a human IEC line (72). However, further studies are needed to determine the specific role of type III IFNs in control of enteroviruses. Canine parvovirus, which causes gastrointestinal disease in dogs, is more sensitive to IFN-λ than a type I IFN *in vitro* (73), but it is unknown whether this applies to human parvoviruses. Porcine epidemic diarrhea virus is an enteropathogenic coronavirus that is sensitive to both type I and III IFN treatment in a porcine IEC line (74). Finally, avian influenza virus and Newcastle disease virus induce much more robust type III than type I IFN in a primary chicken IEC culture model, suggesting a possible role for IFN-λ in prevention of intestinal infection by these viruses normally associated with respiratory infections (75). These initial findings point to the potential for a broad role for IFN-λ in control of many different enteric viruses, but additional studies are clearly needed to determine the breadth and depth of IFN-λ-mediated regulation of viral infection in the intestine.

## IFN-λ Interactions with Other Signaling Pathways

Interferon-lambda-mediated antiviral immunity in the intestine against rotavirus (EDIM-RV) and MNoV does not redundantly overlap with type I IFNs, while there is redundancy between type I and III IFNs to control influenza, SARS coronavirus, and respiratory syncytial virus in the lung, and herpes simplex virus-2 in the genital tract [reviewed in Ref. (76)]. There are two potential reasons for a non-redundant role for IFN-λ in the intestine. First, the IFN-λ receptor is highly expressed in IECs but is minimally detectable in other intestinal cell types such as lamina propria cells (50). Second, expression of IFN-α receptor subunits (i.e., IFNAR1 and IFNAR2) is less abundant in IECs than in lamina propria cells (14), and surface expression of the IFN-α receptor is polarized to the apical side (41). Interestingly, in neonatal mice, IECs are sensitive to both IFN-α/β and IFN-λ, and both IFN-α/β and IFN-λ can control RV (RRV strain) infection in suckling mice (24). It has not been explored whether this IFN-α/β-sensitivity in neonatal IECs is from altered trafficking of the IFN-α receptor to the basolateral side. Further work is needed to explore the consequences of age-related IFN-α/β sensitivity in IECs and the pathogenesis of enteric virus infection (Figure 1).

Another cytokine important for mucosal immunity, IL-22, has a synergistic relationship with IFN-λ. Similar to IFNLR1, the IL-22 receptor subunit, IL22Rα, associates with IL10Rβ and is expressed preferentially by IECs (77). During RV infection, IL-22 acts coordinately with IFN-λ to control virus replication and prevent tissue damage in mice (16). This antiviral activity of IL-22 is *Ifnlr1* and *Stat1* dependent but not *Stat3* dependent. IL-22 also restricts porcine enteric coronavirus infection in the intestine, for which antiviral activity is largely *Stat3* dependent (78). Since IL-22 also induces IFN-λ expression in the intestine, a *Stat3*-independent/IFN-λ-dependent role for IL-22 in control of porcine enteric coronavirus cannot be ruled out (78).

Finally, lactoferrin, a member of the transferrin family and a component of milk, potentiates IFN-λ production in a human IEC line (79), and *in vitro* lactoferrin has antiviral activity against RV (80) and MNoV (81). Thus, it would be interesting to study whether milk-derived components exhibit cross talk with IFN-λ-mediated immunity for enteric viral infections in neonatal hosts.

## IFN-λ and Transkingdom Interactions

A final critical factor for discussion of enteric viral infections and IFN-λ is the role of the commensal bacterial microbiome. For these viruses, infection occurs amidst the complex milieu of the oral and intestinal microbiome, which plays important roles in regulation of viral infectivity. Poliovirus, reovirus, and murine mammary tumor virus depend upon the presence of commensal bacteria for infection (82, 83), with direct viral binding to bacterial products like lipopolysacchide implicated as the mechanism of facilitation (84, 85). Depletion of the commensal microbiota also impairs RV infection and results in enhancement of both mucosal and systemic antibody responses against the virus (86). Human NoV binds directly to bacterial products that mimic the histo-blood group antigens (HBGAs) known to be attachment factors for NoV (87–89), and indeed culture of human NoV in B cells depends on the presence of these HBGA-expressing bacteria (57, 90). Hence, there is a common theme for enteric viruses in interacting with and depending on intestinal bacteria for infectivity, though the specific mechanisms may be virus dependent (91, 92).

The link between viral dependence on the microbiome and sensitivity to IFN-λ comes from work done with MNoV. Depletion of the commensal microbiota in wild-type mice prevents persistent intestinal MNoV infection (93), similar to what has been observed with other enteric viruses. Interestingly, in mice lacking *Ifnlr1, Stat1*, or *Irf3*, all important molecules for IFN-λ induction or signaling, MNoV establishes infection even in the absence of commensal microbes, implicating IFN-λ in regulation of these transkingdom viral–bacterial interactions (93) (Figure 1). Other enteric viruses share both a dependence on the microbiome and a sensitivity to IFN-λ; whether interplay between the microbiome and IFN-λ signaling also regulates other intestinal viruses such as RV and reovirus remains to be seen.

## Gut Instinct about the Future of IFN-λ

While the past decade yielded many exciting insights into regulation of enteric viruses by IFN-λ, many important questions remain. Type I and III IFNs share significant overlap in induction and signaling pathways, though there are distinctions in promoter sequences, upstream regulatory elements, and kinetics of downstream gene stimulation [reviewed in Ref. (76)]. However, most previous studies were performed *in vitro* outside of the complex environment of the gut. How is IFN-λ production regulated in the intestine, and by what pathways is it induced *in vivo* by viral infection? Are specific ISGs induced by IFN-λ necessary for antiviral activity against enteric infections? Conversely, viruses rapidly evolve mechanisms to evade the host immune system. Are there viral factors that specifically target IFN-λ induction or signaling pathways for evasion or suppression?

In addition to important mechanistic questions for enteric viruses already known to be IFN-λ regulated, there are a number of intestinal viruses for which sensitivity to IFN-λ has not yet been explored. Astroviruses, parvoviruses, enteroviruses, and adenoviruses are among the enteric viruses for which data on IFN-λ-sensitivity in both cell culture and animal models is currently lacking. Finally, of great interest is the *in vivo* effect of IFN-λ regulation on enteric viruses in humans. Single-nucleotide polymorphisms (SNPs) in human IFN-λ genes are associated with differential responses to hepatitis B and C, human cytomegalovirus, herpes simplex virus 1, and influenza virus vaccination [reviewed in Ref. (76, 94)]. Enteric infections cause a spectrum of disease in different individuals, including variable severity and duration of infection, which may correlate with host genetic variation. Do these same SNPs correlate with differential responses to enteric viruses or to vaccination? IFN-λ is clearly an important innate immune regulator for many gut viruses, and defining the breadth of its effects and the mechanisms underlying its enteric activity represent exciting areas for future research endeavors.

## Author Contributions

All authors contributed equally to this work. SL and MB conceptualized, wrote, and edited the manuscript.

## Conflict of Interest Statement

The authors declare that the research was conducted in the absence of any commercial or financial relationships that could be construed as a potential conflict of interest. The reviewer, YT, and handling editor declared their shared affiliation, and the handling editor states that the process nevertheless met the standards of a fair and objective review.
